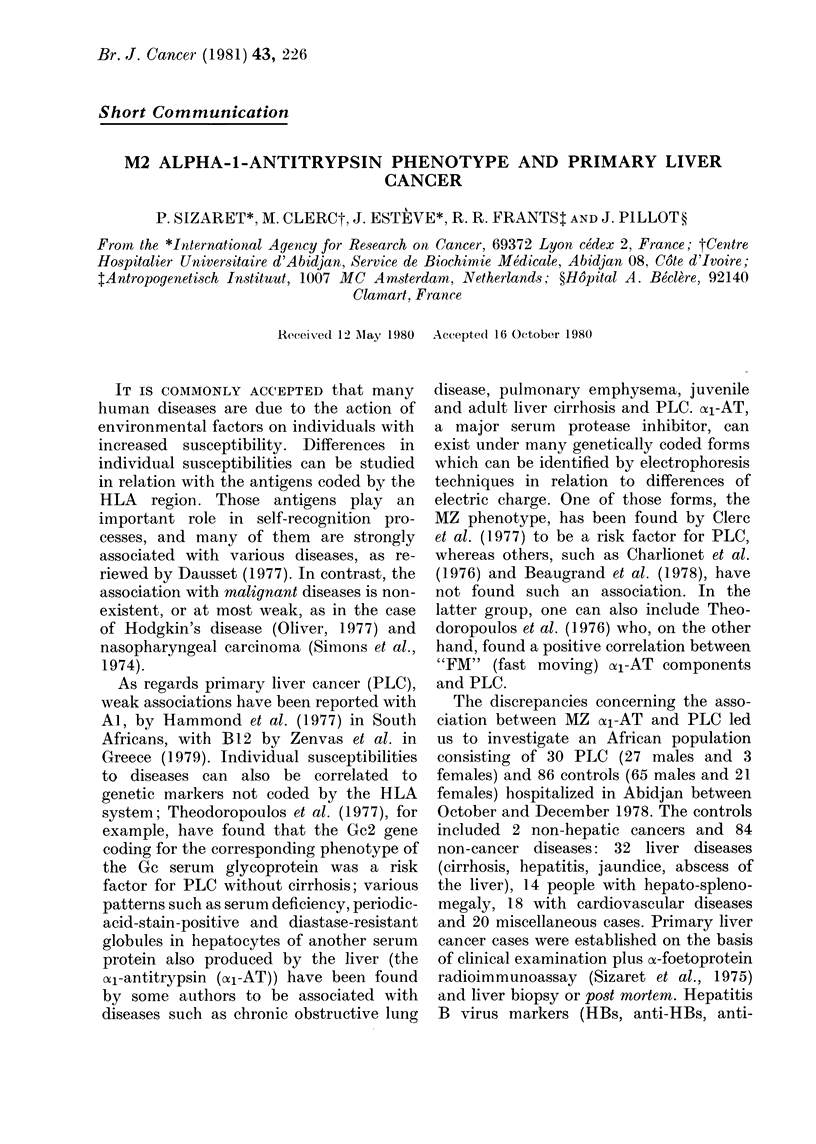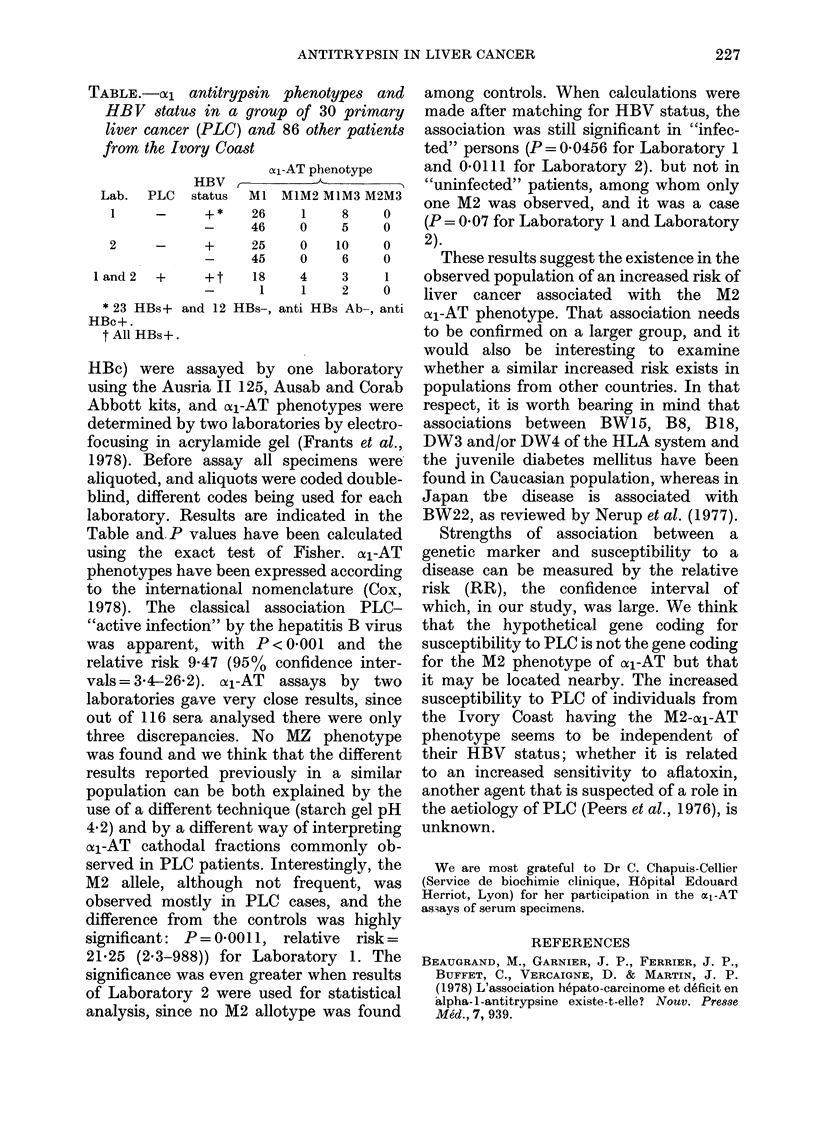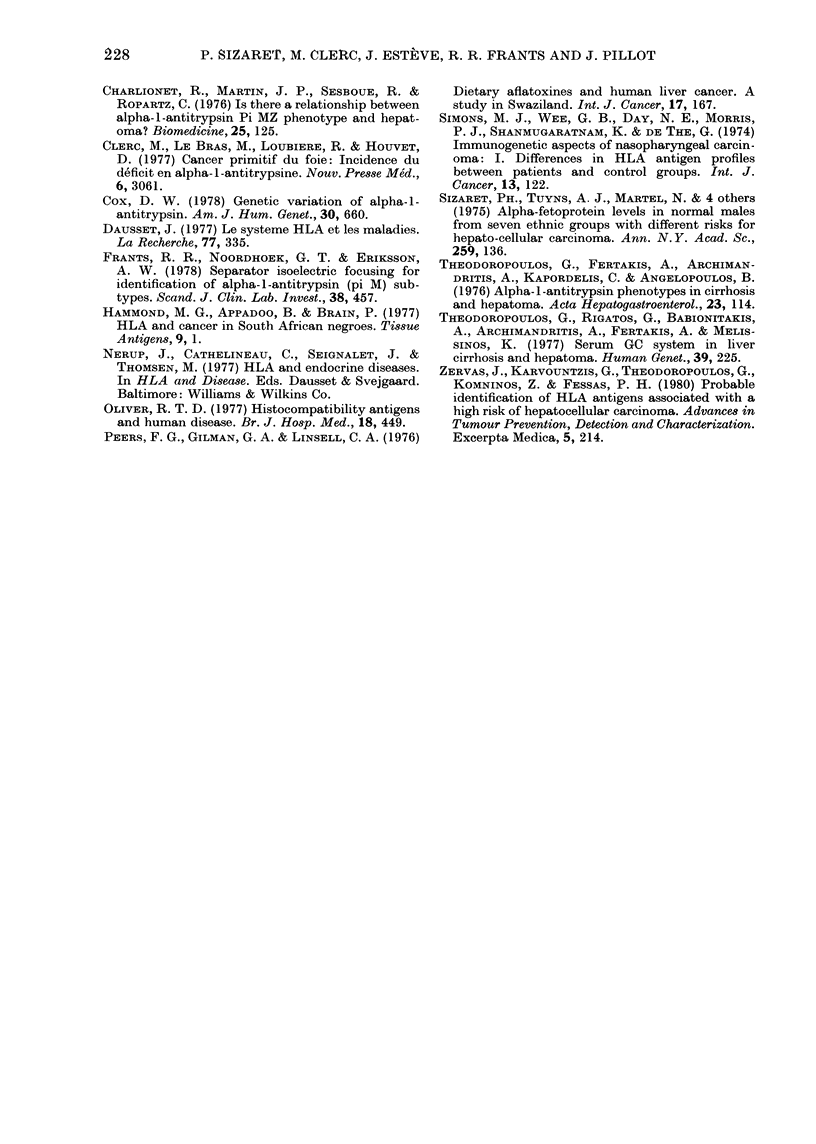# M2 alpha-1-antitrypsin phenotype and primary liver cancer.

**DOI:** 10.1038/bjc.1981.32

**Published:** 1981-02

**Authors:** P. Sizaret, M. Clerc, J. Estève, R. R. Frants, J. Pillot


*Br. J. Cancer* (1981) **43**, 226

### *Short Communication*

### M2 ALPHA-1-ANTITRYPSIN PHENOTYPE AND PRIMARY LIVER CANCER

P. SIZARET*, M. CLERC†, J. ESTÈVE*, R. R. FRANTS‡ AND J. PILLOT§

*From the *International Agency for Research on Cancer, 69372 Lyon cédex 2, France; †Centre Hospitalier Universitaire d'Abidjan, Service de Biochimie Médicale, Abidjan 08, Côte d'Ivoire; ‡Antropogenetisch Instituut, 1007 MC Amsterdam, Netherlands; §Hôpital A. Béclère, 92140 Clamart, France*

Received 12 May 1980 Accepted 16 October 1980

IT IS COMMONLY ACCEPTED that many human diseases are due to the action of environmental factors on individuals with increased susceptibility. Differences in individual susceptibilities can be studied in relation with the antigens coded by the HLA region. Those antigens play an important role in self-recognition processes, and many of them are strongly associated with various diseases, as reriewed by Dausset (1977). In contrast, the association with *malignant* diseases is non-existent, or at most weak, as in the case of Hodgkin's disease (Oliver, 1977) and nasopharyngeal carcinoma (Simons *et al.*, 1974).

As regards primary liver cancer (PLC), weak associations have been reported with A1, by Hammond *et al.* (1977) in South Africans, with B12 by Zenvas *et al.* in Greece (1979). Individual susceptibilities to diseases can also be correlated to genetic markers not coded by the HLA system; Theodoropoulos *et al.* (1977), for example, have found that the Gc2 gene coding for the corresponding phenotype of the Gc serum glycoprotein was a risk factor for PLC without cirrhosis; various patterns such as serum deficiency, periodic-acid-stain-positive and diastase-resistant globules in hepatocytes of another serum protein also produced by the liver (the $\alpha_1$-antitrypsin ($\alpha_1$-AT)) have been found by some authors to be associated with diseases such as chronic obstructive lung disease, pulmonary emphysema, juvenile and adult liver cirrhosis and PLC. $\alpha_1$-AT, a major serum protease inhibitor, can exist under many genetically coded forms which can be identified by electrophoresis techniques in relation to differences of electric charge. One of those forms, the MZ phenotype, has been found by Clerc *et al.* (1977) to be a risk factor for PLC, whereas others, such as Charlionet *et al.* (1976) and Beaugrand *et al.* (1978), have not found such an association. In the latter group, one can also include Theodoropoulos *et al.* (1976) who, on the other hand, found a positive correlation between "FM" (fast moving) $\alpha_1$-AT components and PLC.

The discrepancies concerning the association between MZ $\alpha_1$-AT and PLC led us to investigate an African population consisting of 30 PLC (27 males and 3 females) and 86 controls (65 males and 21 females) hospitalized in Abidjan between October and December 1978. The controls included 2 non-hepatic cancers and 84 non-cancer diseases: 32 liver diseases (cirrhosis, hepatitis, jaundice, abscess of the liver), 14 people with hepato-splenomegaly, 18 with cardiovascular diseases and 20 miscellaneous cases. Primary liver cancer cases were established on the basis of clinical examination plus $\alpha$-foetoprotein radioimmunoassay (Sizaret *et al.*, 1975) and liver biopsy or *post mortem*. Hepatitis B virus markers (HBs, anti-HBs, anti-

TABLE.—$\alpha_1$ *antitrypsin phenotypes and HBV status in a group of* 30 *primary liver cancer (PLC) and* 86 *other patients from the Ivory Coast*

| Lab. | PLC | HBV status | $\alpha_1$-AT phenotype M1 | M1M2 | M1M3 | M2M3 |
|---|---|---|---|---|---|---|
| 1 | − | +* | 26 | 1 | 8 | 0 |
| | | − | 46 | 0 | 5 | 0 |
| 2 | − | + | 25 | 0 | 10 | 0 |
| | | − | 45 | 0 | 6 | 0 |
| 1 and 2 | + | +† | 18 | 4 | 3 | 1 |
| | | − | 1 | 1 | 2 | 0 |

* 23 HBs+ and 12 HBs−, anti HBs Ab−, anti HBc+.

† All HBs+.

HBc) were assayed by one laboratory using the Ausria II 125, Ausab and Corab Abbott kits, and $\alpha_1$-AT phenotypes were determined by two laboratories by electrofocusing in acrylamide gel (Frants *et al.*, 1978). Before assay all specimens were aliquoted, and aliquots were coded double-blind, different codes being used for each laboratory. Results are indicated in the Table and $P$ values have been calculated using the exact test of Fisher. $\alpha_1$-AT phenotypes have been expressed according to the international nomenclature (Cox, 1978). The classical association PLC–"active infection" by the hepatitis B virus was apparent, with $P < 0{\cdot}001$ and the relative risk 9·47 (95% confidence intervals = 3·4–26·2). $\alpha_1$-AT assays by two laboratories gave very close results, since out of 116 sera analysed there were only three discrepancies. No MZ phenotype was found and we think that the different results reported previously in a similar population can be both explained by the use of a different technique (starch gel pH 4·2) and by a different way of interpreting $\alpha_1$-AT cathodal fractions commonly observed in PLC patients. Interestingly, the M2 allele, although not frequent, was observed mostly in PLC cases, and the difference from the controls was highly significant: $P = 0{\cdot}0011$, relative risk = 21·25 (2·3–988)) for Laboratory 1. The significance was even greater when results of Laboratory 2 were used for statistical analysis, since no M2 allotype was found among controls. When calculations were made after matching for HBV status, the association was still significant in "infected" persons ($P = 0{\cdot}0456$ for Laboratory 1 and 0·0111 for Laboratory 2). but not in "uninfected" patients, among whom only one M2 was observed, and it was a case ($P = 0{\cdot}07$ for Laboratory 1 and Laboratory 2).

These results suggest the existence in the observed population of an increased risk of liver cancer associated with the M2 $\alpha_1$-AT phenotype. That association needs to be confirmed on a larger group, and it would also be interesting to examine whether a similar increased risk exists in populations from other countries. In that respect, it is worth bearing in mind that associations between BW15, B8, B18, DW3 and/or DW4 of the HLA system and the juvenile diabetes mellitus have been found in Caucasian population, whereas in Japan the disease is associated with BW22, as reviewed by Nerup *et al.* (1977).

Strengths of association between a genetic marker and susceptibility to a disease can be measured by the relative risk (RR), the confidence interval of which, in our study, was large. We think that the hypothetical gene coding for susceptibility to PLC is not the gene coding for the M2 phenotype of $\alpha_1$-AT but that it may be located nearby. The increased susceptibility to PLC of individuals from the Ivory Coast having the M2-$\alpha_1$-AT phenotype seems to be independent of their HBV status; whether it is related to an increased sensitivity to aflatoxin, another agent that is suspected of a role in the aetiology of PLC (Peers *et al.*, 1976), is unknown.

We are most grateful to Dr C. Chapuis-Cellier (Service de biochimie clinique, Hôpital Edouard Herriot, Lyon) for her participation in the $\alpha_1$-AT assays of serum specimens.

### REFERENCES

BEAUGRAND, M., GARNIER, J. P., FERRIER, J. P., BUFFET, C., VERCAIGNE, D. & MARTIN, J. P. (1978) L'association hépato-carcinome et déficit en alpha-1-antitrypsine existe-t-elle? *Nouv. Presse Méd.*, 7, 939.

CHARLIONET, R., MARTIN, J. P., SESBOUE, R. & ROPARTZ, C. (1976) Is there a relationship between alpha-1-antitrypsin Pi MZ phenotype and hepatoma? *Biomedicine*, **25**, 125.

CLERC, M., LE BRAS, M., LOUBIERE, R. & HOUVET, D. (1977) Cancer primitif du foie: Incidence du déficit en alpha-1-antitrypsine. *Nouv. Presse Méd.*, **6**, 3061.

COX, D. W. (1978) Genetic variation of alpha-1-antitrypsin. *Am. J. Hum. Genet.*, **30**, 660.

DAUSSET, J. (1977) Le systeme HLA et les maladies. *La Recherche*, **77**, 335.

FRANTS, R. R., NOORDHOEK, G. T. & ERIKSSON, A. W. (1978) Separator isoelectric focusing for identification of alpha-1-antitrypsin (pi M) subtypes. *Scand. J. Clin. Lab. Invest.*, **38**, 457.

HAMMOND, M. G., APPADOO, B. & BRAIN, P. (1977) HLA and cancer in South African negroes. *Tissue Antigens*, **9**, 1.

NERUP, J., CATHELINEAU, C., SEIGNALET, J. & THOMSEN, M. (1977) HLA and endocrine diseases. In *HLA and Disease*. Eds. Dausset & Svejgaard. Baltimore: Williams & Wilkins Co.

OLIVER, R. T. D. (1977) Histocompatibility antigens and human disease. *Br. J. Hosp. Med.*, **18**, 449.

PEERS, F. G., GILMAN, G. A. & LINSELL, C. A. (1976) Dietary aflatoxines and human liver cancer. A study in Swaziland. *Int. J. Cancer*, **17**, 167.

SIMONS, M. J., WEE, G. B., DAY, N. E., MORRIS, P. J., SHANMUGARATNAM, K. & DE THE, G. (1974) Immunogenetic aspects of nasopharyngeal carcinoma: I. Differences in HLA antigen profiles between patients and control groups. *Int. J. Cancer*, **13**, 122.

SIZARET, PH., TUYNS, A. J., MARTEL, N. & 4 others (1975) Alpha-fetoprotein levels in normal males from seven ethnic groups with different risks for hepato-cellular carcinoma. *Ann. N.Y. Acad. Sc.*, **259**, 136.

THEODOROPOULOS, G., FERTAKIS, A., ARCHIMANDRITIS, A., KAPORDELIS, C. & ANGELOPOULOS, B. (1976) Alpha-1-antitrypsin phenotypes in cirrhosis and hepatoma. *Acta Hepatogastroenterol.*, **23**, 114.

THEODOROPOULOS, G., RIGATOS, G., BABIONITAKIS, A., ARCHIMANDRITIS, A., FERTAKIS, A. & MELISSINOS, K. (1977) Serum GC system in liver cirrhosis and hepatoma. *Human Genet.*, **39**, 225.

ZERVAS, J., KARVOUNTZIS, G., THEODOROPOULOS, G., KOMNINOS, Z. & FESSAS, P. H. (1980) Probable identification of HLA antigens associated with a high risk of hepatocellular carcinoma. *Advances in Tumour Prevention, Detection and Characterization.* Excerpta Medica, **5**, 214.